# Orodispersible Film Loaded with *Enterococcus faecium* CRL183 Presents Anti-*Candida albicans* Biofilm Activity In Vitro

**DOI:** 10.3390/pharmaceutics13070998

**Published:** 2021-06-30

**Authors:** Virgínia Barreto Lordello, Andréia Bagliotti Meneguin, Sarah Raquel de Annunzio, Maria Pía Taranto, Marlus Chorilli, Carla Raquel Fontana, Daniela Cardoso Umbelino Cavallini

**Affiliations:** 1 School of Pharmaceutical Sciences, Sao Paulo State University (UNESP), Rodovia Araraquara-Jaú, Km 01-s/n, Campus Ville, Araraquara 14800-903, Brazil; virginialordello@gmail.com (V.B.L.); andreia.meneguin@unesp.br (A.B.M.); sarinha_annunzio@hotmail.com (S.R.d.A.); marlus.chorilli@unesp.br (M.C.); 2 Reference Center for Lactobacilli (CERELA-CONICET), San Miguel de Tucumán, Chacabuco 145, Tucumán T4000 ILC, Argentina; ptaranto@cerela.org.ar

**Keywords:** probiotics, biofilm, anti-Candida activity, orodispersible films, *Candida albicans*, *Enterococcus faecium*

## Abstract

Background: Probiotic bacteria have been emerging as a trustworthy choice for the prevention and treatment of *Candida* spp. infections. This study aimed to develop and characterize an orodispersible film (ODF) for delivering the potentially probiotic *Enterococcus faecium* CRL 183 into the oral cavity, evaluating its in vitro antifungal activity against *Candida albicans*. Methods and Results: The ODF was composed by carboxymethylcellulose, gelatin, and potato starch, and its physical, chemical, and mechanical properties were studied. The probiotic resistance and viability during processing and storage were evaluated as well as its in vitro antifungal activity against *C. albicans*. The ODFs were thin, resistant, and flexible, with neutral pH and microbiologically safe. The probiotic resisted the ODF obtaining process, demonstrating high viability (>9 log_10_ CFU·g^−1^), up to 90 days of storage at room temperature. The Probiotic Film promoted 68.9% of reduction in fungal early biofilm and 91.2% in its mature biofilm compared to the group stimulated with the control film. Those results were confirmed through SEM images. Conclusion: The probiotic ODF developed is a promising strategy to prevent oral candidiasis, since it permits the local probiotic delivery, which in turn was able to reduce *C. albicans* biofilm formation.

## 1. Introduction

It is well established that probiotics, which are defined as “living microorganisms that, when administered in adequate amount, confer a health benefits to the host” [1], can improve dysbiotic microbiota, regulate intestinal transit, neutralize carcinogens, promote vitamin synthesis and bile salt metabolism, reinforce gut barrier and the immune system, and directly antagonize and compete with pathogens [2]. Several studies have shown that specific probiotic strains have great potential for improving oral health, presenting anticariogenic activity, reduction of halitosis, and prevention of opportunistic infections, such as candidiasis, which are often present in immunocompromised individuals [3,4,5,6,7].

These pathogenic conditions are related to the formation and establishment of biofilms in the oral cavity. Infections caused by biofilms, especially fungal biofilms, are long-lasting and difficult to eliminate. Therefore, high doses of antifungals are often required, which promote serious adverse effects due to the similarity between host and fungal cells and lead to pharmacotherapy resistance [7,8,9].

Thus, alternative approaches for fungal infections must be searched, and prevention seems to be the best strategy. Our research group has demonstrated that *Enterococcus faecium* CRL 183, a proven safe potentially probiotic strain [10,11], was able to impair *Candida albicans* ATCC 90028 in vitro in up to 99.9% without losing its own viability. Researchers tested the interaction of fungus–probiotic in different proportions (1:1; 1:10, and 1:100), and even in its lowest concentration (10^7^ CFU·mL^−1^), *E. faecium* CRL 183 impaired *C. albicans* viability in 97.12% (results awaiting publication). Those findings agree with several studies that show that *C. albicans* spp. supports the survival of *Enterococcus* spp., and this bacterium attenuates that fungus virulence and viability in polymicrobial infections through the secretion of proteases, bacteriocins, and lactic acid [10,12,13,14,15,16]. *E. faecium* CRL 183 was also able to inhibit the growth of *Streptococcus mutans* ATCC 25175, which is another important pathogen of the oral cavity [17].

One of the greatest challenges concerning probiotic products is to develop non-dairy alternatives focusing on local actions. These novel products should grant probiotic viability during processing and storage, should not alter product flavor or consistency and, on the oral cavity concern, should stay in the mouth long enough to release the probiotic [17,18,19].

An option that meets all these requirements is the orodispersible films (ODFs), which are also known as soluble films, oral disintegrating films, oral thin films or mucoadhesive films—the nomenclature changes depending on their characteristics and functionalities [20]. This innovative product can be defined as a thin film of easy dissolution and high contact surface, which allows the release of all active components into the oral cavity. Among the advantages attributed to these films are storage at room temperature, ease of obtaining, handling, and administration, not requiring water, efficient release of active components, and facilitating patient compliance to treatment. Lastly, as the oral mucosa is highly vascularized, ODF can also avoid the first-pass metabolism [21,22].

Researchers have shown that this pharmaceutical form may be the best strategy to immobilize probiotics and deliver them into specific areas such as the oral cavity. Probiotic ODF presented high microbial viability after processing, allowing a proper shelf-life, suitable disintegration time allowing the local probiotic release, and good appearance and mechanical properties [18,19,23].

Those films are usually composed of macromolecules, natural or synthetic, forming a polymer matrix, plasticizers—which increase the filmogenic properties of the polymers giving greater flexibility and less fragility—and the bioactive compound of interest. The composition and proportion of such excipients can be altered according to the characteristics desired for the product [20]. The chosen polymers should be safe, have a good shelf life, should not be toxic to the probiotic, ensuring its viability and metabolic activity, as well as have a quick and efficient release [19,24]. Carboxymethylcellulose (CMC), hydroxypropyl methylcellulose (HPMC), and gelatin are considered good choices for films that carry probiotics [18,19,22,23]. However, films composed of a CMC and gelatin blend were more efficient at protecting probiotic cells during the drying process of the films, and those composed of CMC, gelatin, and starch allowed the survival of probiotics in a high concentration (>9 CFU·g^−1^) for a longer period [18].

According to the characteristics of the films, these constitute carriers of bioactive substances that are ideal for patients with conditions that favor dysphagia such as the elderly, patients with motor debilities, and the immunocompromised [19,21,22]. Coincidentally, in this population, there is a high prevalence of candidiasis [19,21,22,25]. Considering the above, this study aimed to develop and characterize an ODF with the addition of the probiotic *E. faecium* CRL 183, evaluating its antifungal potential against *C. albicans* ATCC 90028 biofilms in vitro.

## 2. Materials and Methods

The probiotic strains *E. faecium* CRL 183 and *C. albicans* ATCC 90028 were obtained from the Reference Center for Lactobacilli—CERELA/CONICET (San Miguel de Tucumán, Argentina) and from American Type Culture Collection (Manassas, VA, USA), respectively. CMC was donated by Biovital (São Carlos, SP, Brazil), gelatine (260 bloom/30 mesh) was provided by Gelita (Cotia, SP, Brazil), sorbitol (Neosorb P60W) was supplied by Labonathus (São Paulo, SP, Brazil), alcohol-free mint flavoring was provided by Firmenich, (Cotia, SP, Brazil), and potato starch was purchased from Yoki (Pouso Alegre, MG, Brazil). 

### 2.1. Strains and Growth Conditions

The strains were thawed and subcultured in specific culture media: *C. albicans* in Sabouraud Dextrose Agar supplemented with chloramphenicol (0.05 g·L^−1^) (SDA—Acumedia, Lansing, MI, USA) and *E. faecium* in Bile Esculin Agar or M17 agar (Acumedia, Lansing MI, USA) and incubated at 37 °C for 48 h. *C. albicans* colonies, freshly cultivated as described above, were inoculated with a sterile loop in enriched broth (26 g·L^−1^ of BHI—Kasvi, Curitiba, Brazil; 10 g·L^−1^ of YE—Acumedia, Lansing MI, USA; 20 g·L^−1^ of TSB—Acumedia, Lansing, MI, USA; 20% of sucrose Synth, Diadema, Brazil). This yeast suspension was standardized (6 log_10_ CFU·mL^−1^) by spectrophotometry reading on a Synergy H1M microplate reader (Biotek, Winooski, VT, USA). This initial concentration was based on previous study, which has shown that in our experimental design, starting the *Candida albicans* biofilm with an initial concentration of 6 log_10_ CFU·mL^−1^ or 8 log_10_ CFU·mL^−1^ did not result in a statistically significant difference in the final population of microorganisms in the biofilms (results awaiting publication).

To prepare the probiotic inoculum, *E. faecium* freshly cultivated colonies were inoculated in 5 mL of M17 Broth (Himedia, Mumbai, India) followed by incubation at 35 ± 2 °C for 14–16 h. After this period, the tubes containing the inoculum were pelleted by centrifuging at 1431 g (80-2B Centribio-Equipar, Curitiba, Brazil) for 20 min, washed twice with 0.9% (*w*/*v*) NaCl solution (Synth, Diadema, Brazil), and resuspended in a lower volume of 0.9% (*w*/*v*) NaCl. The bacterial suspension obtained was standardized at ≈10 log_10_ CFU·mL^−1^ [18].

### 2.2. Orodispersible Films Preparation

**Figure 1:**
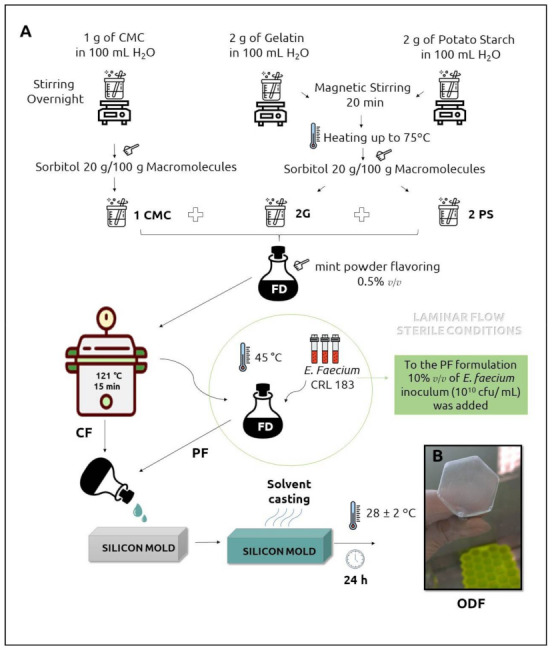
Orodispersible films preparation protocol (**A**). Orodispersible film (**B**).

Two formulations of ODFs—Probiotic Film (PF) and Control Film (CF)—were prepared by solvent casting as described by Heinemann et al. [18], with modifications. The procedure is outlined in Figure 1 [18]. Three dispersions of macromolecules (polymers) and plasticizer were prepared using a magnetic stirrer (Fisatom, São Paulo, SP, Brazil): G—2.00 g gelatin + 0.4 g sorbitol in 100 mL distilled water; PS—2.00 g potato starch + 0.4 g sorbitol in 100 mL distilled water, and CMC—1.00 g CMC + 0.2 g sorbitol in 100 mL distilled water. These dispersions were mixed in the proportion 1CMC:2PS:2G (*v*/*v*) to obtain the film-forming dispersion (FD), to which 0.50% (*w*/*w*) mint powder flavoring was added. The film-forming dispersion was sterilized at 121 °C/15 min and then cooled to 45 °C. After that, all the steps were conducted within the laminar flow under sterile conditions. To prepare the PF, 10% (*v*/*v*) of the freshly cultured *E. faecium* CRL 183 inoculum was added to FD (45 °C) under constant agitation. Two milliliters of FD were poured into 2 × 2 cm holes of silicone molds to obtain films with standardized size and weight. The silicone molds were arranged in laminar flows with constant air circulation at room temperature (28 ± 2 °C) for 24 h. The obtained films were carefully removed from the silicone molds and conditioned in Petri dishes at room temperature within a vacuum desiccator. The films were produced in three batches. The same procedure was used to obtain CF, without the step of adding *E. faecium* CRL 183 inoculum.

### 2.3. Macroscopic Observations, Color Parameters, Thickness Analysis, pH, and Moisture Content of ODFs

The films were inspected regarding to appearance, texture, flexibility, and occurrence of bubbles and fissures. A colorimeter (Konica Minolta^®^ Spectrophotometer CM 600d, Osaka, Kansai, Japan) was used for the assessment of color parameters (CIELAB system expressed as L*, a* and b* values), in triplicate. The thickness was evaluated with a digital micrometer MDC-25SX (Mitutoyo^®^, Kawasaki, Japan) in three random positions of each film, in sextuplicate. The pH of the films was determined using a digital potentiometer (Qualxtron^®^, Model 8010, Jundiaí, Brazil), through the dissolution of a 0.1 g sample of the films in 20.0 mL of 0.9% (*w*/*v*) NaCl, in triplicate. The moisture content was measured in an infrared analyzer (Sartorius^®^ MA35, Goettingen, Germany), right after the end of the drying period of the films (T0). 

### 2.4. Probiotic Survival in PF after Drying Process and Storage

To evaluate the cell viability of *E. faecium* in the ODFs, PF samples (0.1 g) were solubilized in 20 mL of 0.9% (*w*/*v*) NaCl (Synth, Diadema, Brazil) and serially diluted and plated (spread-plate) in M17 agar (Himedia, Mumbai, India). The results were expressed as log_10_ CFU·g^−1^ after incubation under aerobic conditions at 37 °C/48 h. The analyses were performed immediately after the drying process of ODF (T0) and compared to the probiotic amount added to the filmogenic solution, to evaluate the probiotic resistance to processing. To evaluate the probiotic viability over time, the analysis was performed fortnightly, during the storage period of 90 days.

### 2.5. Microbiological Safety of ODFs

The films’ water activity (Aw) was measured using an Aqualab^®^ CX2 analyzer (Decagon devices, USA) after stabilization of 0.1 g samples at 25 °C for 30 min. The microbiological safety of ODFs was determined monthly through the dissolution of a 0.1 g sample in 20.0 mL of sterile NaCl 0.9% (*w*/*v*). Then, this solution was serial diluted and plated on specific culture media to evaluate contamination by yeasts and molds (PDA—Potato Dextrose Agar, Himedia, India, incubated at 30.0 °C, for 120 h) and total coliforms (Petrifilm^®^ 3M^®^, USA, incubated at 37.0 °C, for 48 h).

### 2.6. In Vitro Disintegration Time

PF or CF samples were placed at the top of a 5 mL Erlenmeyer flask, into which was placed a filter paper. Two hundred microliters of artificial saliva (at 37 °C) prepared according to Nalluri et al. [26] were placed on the films’ surface. The time taken for the drop to pass through the film (T1) or to dissolve the film and form a hole in it (T2) was recorded. The tests were performed at least in triplicate.

### 2.7. Liquid Uptake

An adapted Enslin device [27,28] was used to measure the liquid uptaking ability of the ODFs. Samples of the films were weighted and placed at the bottom of the sintered glass filter of the funnel. The changes on artificial saliva volume on the graduated pipette of the device due to sample absorption were measured at 1, 2, 5, 10, 15, 20, and 30 min. The tests were performed in triplicate, and the results were expressed as medium absorbed (%) in relation to the initial mass of the sample as a function of time.

### 2.8. Water Vapor Permeability (WVP)

PF and CF samples were cut in circular sections and fixed at the top of glass cups with 1.1 cm openings. The cups were previously filled with 10 mL of water (100% relative humidity gradient at 25 °C). The set was accurately weighed and stored in a vacuum desiccator containing silica gel. Therefore, the relative humidity inside the cell was always higher than in the outside, and the water vapor transport was determined from the set mass loss. 

The set weight was measured after 0, 24, 48, 72, and 96 h, and the mass changes were plotted as a time function. The slope of each line was calculated by linear regression (r^2^ > 0.99), and the water vapor transmission rate (*WVTR*) was measured from the slope of the straight line (g·h^−1^) by the test area (m^2^). All values of *WVTR* were corrected for the concentration gradient effect in the stagnant air gap inside the cup as suggested by Gennadios et al. [29] Subsequently to the permeation tests, WVP (g·mm·m^−2^·h^−1^·Pa^−1^) was calculated using Equation (1):

(1) $$\mathrm{WVP}=\frac{WVTR_{c}\times L}{∆P}$$

where *WVTRc*: corrected water vapor transmission rate (g·m^−2^·h^−1^); *L*: average film thickness (mm); ∆*P*: water vapor pressure difference between dry atmosphere and pure water.

### 2.9. Mechanical Properties of ODFs

The mechanical properties of CF and PF were evaluated on a texture analyzer TA-XT2 (Stable Micro Systems, Haslemere, Surrey, South East, United Kingdom), using a spherical-ended puncture probe (5 mm). The films’ sections were fixed on a metallic holder with a circular hole (D = 10 mm), and the probe was moved down at 1 mm·s^−1^ with constant velocity (0.1 mm·s^−1^). The trigger force was 0.005 kg, and force versus displacement curves were recorded until the film rupture and used to determine the puncture strength (Ps) and elongation at break (E_b_%) parameters, according to the following Equations (2) and (3) [30].

(2) $$\mathrm{Ps}=\frac{F}{A}$$

where *F* (N): force required to rupture the film; *A* (m^2^): sectional area of the film (*A* = 2*r*·h, where *r* is the hole radius and h is the film thickness).

(3) $$E_{b}\%=\frac{\sqrt{r^{2}+d^{2}}-r}{r}\times 100$$

where *r* (mm): radius of the exposed film on the orifice plate and *d* is the displacement.

### 2.10. Mucoadhesive Force

The mucoadhesive strength of PF and CF was evaluated by measuring the force required to detach the ODFs from a mucin disc using a TA-XT2 Texture Analyzer (Stable Micro Systems) [31]. Mucin discs were hydrated with artificial saliva (25 µL) and placed on a support for mucoadhesion test. ODFs were attached with double-sided tape to the analytical probe (10 mm), which was then lowered (10 mm·min^−1^) until the mucin disc was in contact with the surface of the films. The complex mucin disc films were kept in contact for 60 s, and then, the probe was moved up at a constant speed (20 mm·min^−1^). The maximum force needed to detach the films from mucin discs, mucoadhesive force (FMA), was recorded.

### 2.11. Evaluation of the Probiotic Film (PF) Anti-Candida albicans Activity

To evaluate whether PF would prevent *C. albicans* biofilm formation and maturation, biofilm assays were carried out following the methods described by Fontana et al. [32] and Zago et al. [33], with modifications. The assays were conducted on 6-well microplates with each well containing 2 mL of standard yeast inoculum (prepared as described in strains and growth conditions) plus 2 mL of enriched broth, i.e., 4 mL per well. In the test group, a sample of 0.1 g of PF (containing approximately 9 log_10_ CFU·g^−1^ *E. faecium*) was added to each well immediately afterwards. To the control group, 0.1 g of CF was added to the wells immediately afterwards. The microplates were incubated at 35 ± 2 °C for 24 h to evaluate the action of the probiotic film in *C. albicans* biofilm adhesion and formation and for 48 h to evaluate it in the biofilm maturation. After that, the supernatant of the biofilms was carefully removed, and its pH was assessed with pH-fix (Neumann-Neander, Düren, Germany), and the adhered biofilms were scraped off the wells and resuspended vigorously in 4 mL of enriched broth with a sterile pipette tip for 30 s. Serial decimal dilutions of those suspensions were made using as diluent the culture medium itself. Microorganisms were quantified by plating in SDA supplemented with chloramphenicol. The plates were incubated for 24 h at 35 ± 2 °C, and the number of fungal cells present in the biofilms was expressed in CFU·mL^−1^. The experiments were performed in triplicate and repeated in three independent assays.

The decimal reduction (DR) of *Candida albicans* cell viability in the presence of PF was determined by Equation (4) [34]:

DR = Log_10_ (Ca_CF_) − Log_10_ (Ca_PF_) (4)

where Ca_CF_: CFU·mL^−1^ values of *C. albicans* present in the biofilms stimulated with control films (CF); Ca_PF_: CFU·mL^−1^ values of *C. albicans* present in the biofilms stimulated with probiotic films (PF).

Then, the percentual reduction (PR) of fungal population after 24 or 48 h was calculated with Equation (5) [34]:

PR = (1 − 10^−DR^) × 100 (5)

where DR is the decimal reduction obtained by Equation (4).

### 2.12. Field Emission Gun Scanning Electron Microscopy (FEG-SEM)

PF and CF surface and transversal section morphology as well as the interactions between *C. albicans* and PF or CF in the early and mature biofilms were assessed by FEG-SEM (JEOL JSM-7500F, Tokyo, Japan). Biofilms were formed as described above in a sterile coverslip placed at the bottom of 6-well microtiter plates. After the incubation period (24 h or 48 h), the biofilms’ supernatants were gently removed, and the coverslip was washed with PBS. The biofilms were fixed with a 2.5% glutaraldehyde solution (Merck, Darmstadt, Germany) and then dehydrated with an ethanol series (30%, 50%, 70%, 85%, and 95% ethanol solution for 15 min each; two washes with 100% ethanol for 15 min). The samples were dried at 37 °C and kept in a vacuum desiccator until the analysis day. For cross-section observations, PF and CF samples were cryofractured by immersion in liquid nitrogen. PF, CF, or the coverslips containing adhered biofilms were attached to the slab surfaces with double-sided adhesive tape and then coated with a layer of carbon and observed through FEG- SEM (JEOL JSM-7500F, Tokyo, Japan).

### 2.13. Statistical Analysis

To verify the statistical significance, the results were submitted to one-way ANOVA followed by Tukey’s multiple comparisons test or Student’s *t*-test (for parametric data) and the Mann–Whitney test (for non-parametric data). It was performed using GraphPad Prism version 7.00 for Windows (GraphPad Software, La Jolla, CA, USA) with a minimum significance level of 5% (*p* ≤ 0.05).

## 3. Results

### 3.1. Macroscopic Observations, Color Parameters, Thickness, pH, Moisture, and Water Activity (Aw) of ODFs

The physicochemical characteristics of ODFs are listed in Table 1. Both CF and PF were macroscopically homogeneous, and without bubbles or fissures (Figure 1B). The incorporation of probiotic increased the thickness, moisture, Aw, and the b* color parameter coordinate (from blue to yellow) (*p* < 0.0001) of ODF. The pH of PF was slightly lower than CF (*p* < 0.03).

### 3.2. Probiotic Survival and Microbiological Safety 

**Figure 2:**
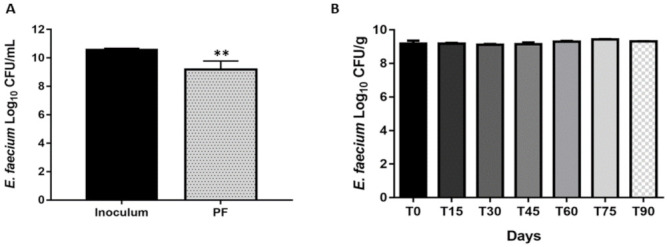
Probiotic resistance. (**A**) Probiotic viability in the inoculum and in PF (Probiotic Film) after drying, according to *t*-test (** *p* = 0.002). (**B**) Probiotic viability during storage, according to ANOVA followed by Tukey’s test.

We observed a reduction in the *E. faecium* CRL183 viability after the drying process was applied to the film production (Figure 2A). However, probiotic cell viability was 9.19 ± 0.58 log_10_ CFU·g^−1^ right after the drying process and remained stable (>9 log_10_ CFU·g^−1^) up to 90 days after the films were formulated (Figure 2B). The ODFs were considered safe, since the population of yeasts and molds and coliforms were less than 1 log_10_ CFU·g^−1^.

### 3.3. In Vitro Disintegration Time and Liquid Uptake

**Figure 3:**
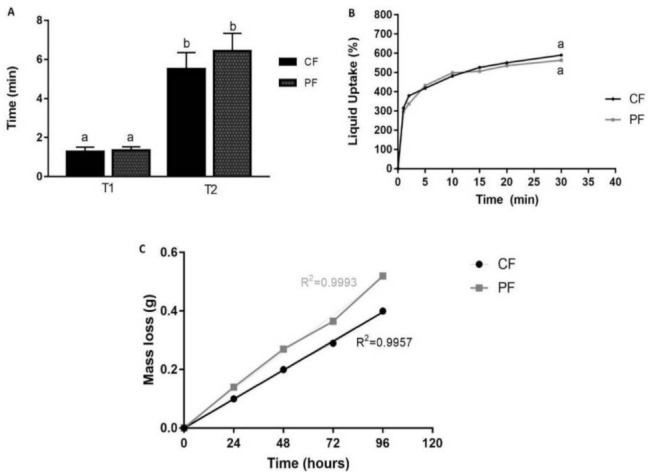
Disintegration time of ODFs; (**A**) T1: time in which artificial saliva cross the films; T2: time to complete disintegration (appearance of a hole). (**B**) CF and PF uptake of artificial saliva over time. (**C**) Mass loss in water vapor permeability test over time. Same lowercase letters show statistical equality according to *t*-test.

There was no difference between the disintegration time for PF (T1: 1.35 min; T2: 6.43 min) and CF (T1:1.28 min; T2: 5.50 min) (Figure 3A). Either PF and CF presented a high and fast ability of artificial saliva uptake (i.e., PF: 297%; CF: 315%) after only 1 min, reaching approximately 600% after 30 min of analysis (*p* > 0.05) (Figure 3B). The mass loss was the same for both ODFs (*p* > 0.05) (Figure 3C).

### 3.4. Mechanical Properties, Water Vapor Permeability (WVP), and Mucoadhesive Force

The probiotic incorporation into the matrix enhanced significantly (*p* = 0.01) WVP and elongation at break values, which can be explained by the greater thickness in these films. The CF and PF were considerably resistant, and the probiotic incorporation did not alter the puncture strength (Ps) and mucoadhesive (FMA) force of the films (Table 2). 

### 3.5. Evaluation of the Probiotic Film (PF) Anti-Candida albicans Activity

**Figure 4:**
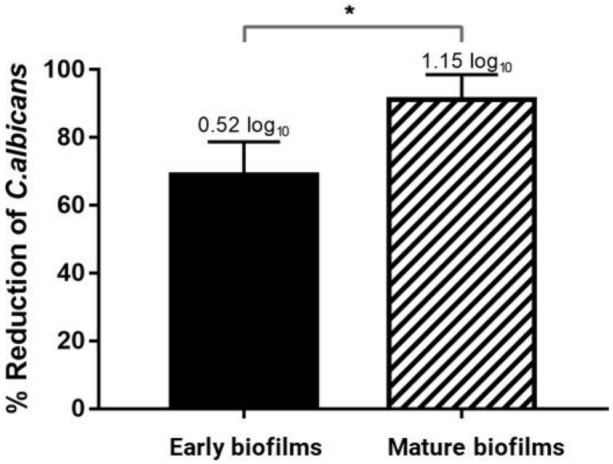
Percentual reduction of *C. albicans* early and mature biofilms biofilm in the presence of PF (Probiotic Film). Each column represents the mean of three independent experiments, each performed in triplicate (*n* = 9), the bars represent the standard deviation. Numbers above columns represent the decimal reduction (DR) The asterisk indicates statistically significant difference (* *p* = 0.03) between early and mature biofilms, according to *t*-test.

The probiotic was efficiently released from the polymeric film, and it impaired in 68.9% (DR = 0.52 log_10_) of the *C. albicans* early biofilm and in 91.24% (DR = 1.15 log_10_) of the the fungal mature biofilm compared to the group stimulated with CF (Figure 4).

### 3.6. Field Emission Gun Scanning Electron Microscopy (FEG-SEM)

**Figure 5:**
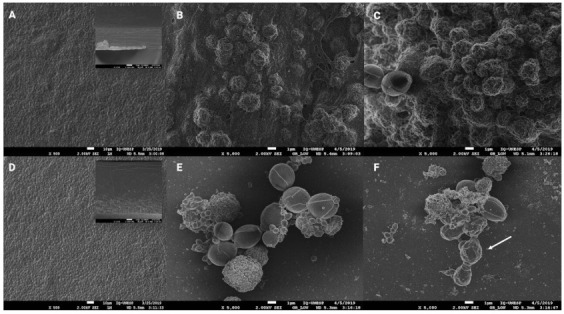
FEG-SEM images. Surface of the ODFs: (**A**) CF 500× and (**D**) PF 500×. The respective cross-sections of each ODF were taken at 1000× and are embedded into the right-hand corner of (**A**,**D**). Biofilms at 5000× magnification: *C. albicans* + CF early (**B**); *C. albicans* + CF mature (**C**); *C. albicans* + PF early (**E**); *C. albicans* + PF mature (**F**). The arrow indicates the wrinkled morphology of *C. albicans* after 48 h in the presence of PF.

The photomicrographs shown in Figure 5 reveal that both CF (Figure 5A) and PF (Figure 5D) present the whole surface without fissures in their structure. Both films’ surfaces were very rough, and the cross-sections SEM images confirmed that PF was thicker than CF.

The photomicrographs of *C. albicans* biofilms stimulated with PF (Figure 5E,F) show free probiotic cells, reassuring the effective release of the probiotic from the polymeric matrix. The SEM images also confirmed the results discussed in section *Evaluation of the Probiotic film (PF) anti-Candida activity*, demonstrating that there was an alteration in the morphology of *C. albicans*, suggesting cell injury in the presence of PF (Figure 5E,F) if compared with the CF group (Figure 5B,C). Furthermore, the biofilms stimulated with CF presented *C. albicans* cells surrounded by a very thick matrix, which occurs in a lesser amount in the biofilms stimulated with PF (Figure 5E,F). In the mature biofilms (Figure 5C,F), it is also possible to notice that yeast cells presented a wrinkled morphology (indicated by the arrow) only when stimulated with PF (Figure 5F), which may indicate that *E. faecium* promotes yeast cell injury, preventing yeast growth and even leading to its death. Future studies have to be conducted to evaluate changes in the expression levels of genes encoding key role proteins for *C. albicans* adhesion during biofilm development (ALS1 and ALS3).

## 4. Discussion

The *E. faecium* CRL 183 strain has beneficial effects in in vitro and in vivo models [17,35,36,37,38]. In this study, we demonstrated that the same strain reduces the biofilm formation of *Candida albicans* when carried in ODFs, without impairing the characteristics of this pharmaceutical form.

Regarding the physicochemical characteristics of ODFs, PF thickness, moisture, and Aw were expected to be higher once there was an incorporation of a great amount (10^10^ CFU·mL^−1^) of probiotic into the polymeric matrix. The residual moisture content in CF was remarkably similar to the results obtained by Heinemann et al. [24], in a formulation also composed by CMC, gelatin, and starch. However, the optimal residual moisture content to maximize the stability of the probiotic varies with the composition of the medium in which it was dehydrated, the storage atmosphere, and the strain, and therefore, it must be determined in each situation [23].

Water activity is the main intrinsic factor related to its physicochemical and microbiological deterioration. Aw values lower than 0.6, similar to those that found in this study, are unfavorable to the growth of contaminating and pathogenic microorganisms while favoring the cellular viability of microorganisms intentionally incorporated into products [17,39]. This is in accordance with the microbiological safety results obtained in this study, as the PF and CF did not show contamination by yeasts and molds or total coliforms in all analyzed times. The ODFs were slightly opaque, and the increase in “b” color parameter of PF was expected once the probiotics inoculum had a cream color, promoting this increase (*p* < 0.0001) in the yellow parameter compared to CF. The difference between the pH (*p* < 0.003) between formulations can be explained by the production of lactic acid by *E. faecium* [40], which decreases the PF pH.

The loss in *E. faecium* cell viability during the processing of the films developed in the present study was 1.24 ± 0.15 log_10_ CFU·g^−1^ (Figure 2A). The viability reduction is expected, since the inoculum was diluted in the polymeric matrix and was exposed to mechanical stress and oxygen during the production of ODFs. Other studies have found a similar reduction in the viability of *Lactobacillus acidophilus* and *Bifidobacterium animalis* subsp. lactis loaded in films composed of CMC, gelatin, and starch [18] or the strain *Bifidobacterium breve* NCIMB 8807 incorporated into a polymeric matrix composed of hydroxypropyl methylcellulose [23].

Concerning the viability during storage (90 days), the population of *E. faecium* in PF was high (<9log_10_ CFU·g^−1^) and constant. Heinemann et al. [18] also verified that *Bifidobacterium animalis* subsp. lactis and *Lactobacillus acidophilus* loaded in ODF (CMC–cellulose/gelatin/starch) remained viable for 2 months at room temperature. However, after 90 days, the microbial counts were heterogeneous, mainly for ODFs under vacuum package [18]. Highlighting that in a previous study, Witzler et al. [17] failed to incorporate *E. faecium* CRL 183 microencapsulated in lozanges, given that its viability decreased from 8.73 log_10_ CFU·g^−1^ to 5.53 log_10_ CFU·g^−1^ shortly after its incorporation, reaching only 1.86 log_10_ CFU·g^−1^ after 28 days of storage. Therefore, the ODF can be a simple and inexpensive way of protection and delivery of *E. faecium* CRL 183, since they are composed by simple and cheap polymers, and the drying process can be performed at room temperature [19,23].

When in contact with biological fluids, ODFs prepared with water-soluble polymers absorb water, leading to volume expansion (swelling) and disintegration. The speed of this process is closely related to the concentration of polymers (the higher, the slower the rate of disintegration) and the interaction of the bioactive compound with the polymer. In this test, it was possible to observe that the films in contact with the artificial saliva quickly swell and form a hydrogel, allowing part of the volume of the saliva to pass through the film (T1), and later, there is the appearance of an orifice (T2) in the films, characterizing the complete disintegration, as described by Garsuch and Breitkreutz [41].

The literature and pharmacopeias do not establish an official threshold for the endpoint of in vitro disintegration tests, stating only that ODF should disperse rapidly [19,21,41]. The T1 observed (PF: 1.35 min and CF 1.28 min) was in accordance with the European Pharmacopeia (2013) [42] orientation for the disintegration threshold for orodispersible tablets (ODTs), which should be within three minutes (180 s). Furthermore, there was an instant swell of the films resulting in a hydrogel which, with the mouth movements, would have been disintegrated [43]. Considering the complete disintegration and appearance of a hole (T2), Heinemann et al. [24] suggested the average time of four minutes as the ideal for the in vitro disintegration of ODF, since this time allows the gradual release of bioactive compounds incorporated into the polymer matrix, without causing fatigue to the consumer. In this same study, the mean in vitro disintegration time was (6.50 ± 0.67 min), which is very similar to the T2 obtained in our study (PF: 6.43 min and CF 5.5 min), for an ODF also composed of CMC, gelatin, and starch [24]. Our results are also in the same time interval of other formulations composed by CMC [24] or gelatin and hydrolyzed collagen [22].

There is a correlation between liquid uptake and the effective release of active pharmaceutical ingredient (API) or bioactive compounds from drug delivery systems [44]. The liquid uptake can also predict the enzymatic degradation of the polymeric matrix and consequently the release of API [27]. CP and PF showed high saliva absorption, and the incorporation of the probiotic did not significantly alter the liquid uptake ability of the films. This was expected once the films were composed of high hydrophilic compounds such as CMC and starch [24,27]. CMC contains hydroxy and carboxyl groups, for which pH values around 7.0 promote the ionization and consequent repulsion of chains with network dilation, favoring the water entrance. Furthermore, the entanglement level of the structures can also play a role in the high liquid uptake presented by CF and PF. The low concentration of polymers in PF and CF composition promotes a looser network that favors water molecule diffusion, compared to stronger structures [28] (Figure 3B). The higher polymeric mass promotes more water absorption [27,28]. Although the addition of new components to polymeric films generally causes the increase in the diffusional path and the barrier properties [45], it was observed that the probiotics incorporation caused the rise of the vapor permeability’s rates. This behavior may be related to the higher moisture content, giving the films a greater affinity for water.

The resistance to cracking during processing, transport, and storage is an important characteristic of polymeric films, and it is determined by its mechanical properties [27]. Elongation at break (Eb%) determines the maximum deformation that a material can undergo before it cracks and, for oral applications, films must be flexible enough to comfortably follow the movement of the mouth [46]. PF was twice as flexible as CF, which can be explained by the residual moisture difference between the two polymeric films; water can act as a plasticizer, intercalating with the polymer chains and therefore enhancing flexibility [21]. Furthermore, loaded films can promote a rearrangement in the polymeric microstructure in a more flexible way [30].

Although mucoadhesive systems can promote a higher residence time in the biological target site, increasing the bioavailability of the active compound [47], the mucoadhesive force of PF and CF was surprisingly low. It is known that mucoadhesive properties can vary with environment-related factors such as pH, contact time, swelling, and physiological characteristics [47]. In this specific case, the fast swellings of the films followed by their disintegration are mechanisms that oppose the establishment of mucoadhesive interactions.

It was determined by our research group that *E. faecium* CRL 183 free cells were able to impair *C. albicans* biofilm formation and maturation in up to 99.9% and 99.43%, respectively, without losing its own viability (data not shown). Among the inhibitory mechanisms exerted by *Enterococcus* spp. on *Candida* spp. Biofilms, the following stand out: competition for nutrients and space, culture media acidification, and secretion of anti-*Candida* peptides, proteases, and other metabolites [9,14,15,16].

Graham et al. [16] described that the enterocin EntV, which is encoded by a gene present in all strains of *E. faecalis* sequenced to date, were able to inhibit several virulence factors of *C. albicans* in addition to decreasing the fungal load and increasing the immune response in a murine model of oral candidiasis. However, on our previous study, it was verified that metabolites produced by *E. faecium* CRL 183 did not exert a fundamental role in its anti-*Candida* activity, and the acidification of the environment and physical competition seems to be the mechanisms by which this antagonism occurs (results awaiting publication). Those anti-Candida strategies are also used for several other probiotic strains [6,48,49,50].

It is known that the optimal pH to *C. albicans* spp. virulence expression is neutral to alkaline [51]. The pH of biofilms (early and mature) stimulated with PF were around 5.0 and with CF were around 7.0, which indicates the production of acid by the probiotic, which was expected since *E. faecium* CRL 183 is a lactic acid bacterium (LAB) [11]. This acidification in the environment could have promoted the yeast cell energy exhaustion, inhibiting its multiplication and leading to cell death [5]. Both competition for nutrients and space and yeast cell exhaustion are possible mechanisms related to the reduction of *C. albicans* biofilms when exposed to PF.

The lesser percentage reduction in the stimuli with PF compared to the stimuli with free cells of the previous study (data not shown) was expected once the probiotic must be firstly released from the polymeric matrix in which it is entrapped in a latent phase without metabolic activity [23]. However, in the context of candidiasis prevention, the results obtained in the present study are very promising, since the *C. albicans* viability reductions were similar to results obtained by other studies with different probiotic strains as free cells [48,49,50]. Probiotic free cells applied into the oral cavity could be removed by the salivary flow within seconds, and therefore, the probiotic would not be able to establish itself in this environment. Moreover, it could present unpleasant sensory properties. Therefore, the present study demonstrates that the ODF containing *E. faecium* CRL 183 may be a more advantageous prevention strategy, since it permits local probiotic release in an adequate amount of time with a good ability to impair *C. albicans* biofilm formation and maturation.

## 5. Conclusions

In conclusion, the PF developed is a promising strategy to prevent oral candidiasis, since it permits the *E. faecium* CRL 183 local delivery which, in its turn, was able to reduce *C. albicans* biofilm formation (68%) and maturation (91%) in vitro. Furthermore, the ODF made of cheap polymers and with a remarkably simple technique was able to maintain high and long-lasting probiotic viability (9 log_10_ CFU·g^−1^ up to 90 days). In addition, it was a suitable way to protect this microorganism during storage, as shown by its good mechanical and barrier properties. Nevertheless, the film was flexible and presented great liquid uptake ability, efficiently releasing the probiotic from a polymeric matrix within a few minutes. These results point the way for future preclinical and clinical studies, which may confirm the anti-*Candida albicans* potential of this probiotic film.

## Figures and Tables

**Table 1:** Physicochemical characterization of ODFs.

	Thickness (mm)	Color (L*, a*, b*)	Moisture (%)	pH	Aw
CF	0.080 ± 0.001 ^A^	L* = 33.53 ± 1.67 a* = −0.24 ± 0.13 b* = −0.49 ± 0.26 ^a^	12.57 ± 0.14 ^A^	6.94 ± 0.003 ^A^	0.378 ± 0.04 ^A^
PF	0.100 ± 0.001 ^B^	L* = 34.28 ± 1.12 a* = −0.22 ± 0.04 b* = +0.80 ± 0.16 ^b^	15.41 ± 0.26 ^B^	6.91 ± 0.005 ^B^	0.404 ± 0.05 ^B^

Means ± SD. Means in the same column followed by different capital letters (A or B) are significantly different by *t*-test (*p* < 0.0001 for thickness, color, moisture, and Aw; *p* < 0.003 for pH). Means in the same column followed by different lowercase letters (a or b) are significantly different by Mann–Whitney test (*p* < 0.0001).

**Table 2:** Mechanical properties, water vapor permeability (WVP), and mucoadhesive force of PF and CF.

Films	WVP (×10^−5^ g mm m^−2^ h^−1^ Pa^−1^)	Ps (MPa)	Eb (%)	FMA (*n*)
CF	1.27 ± 0.1 ^A^	25.61 ± 2.2 ^A^	4.13 ± 0.87 ^A^	0.060 ± 0.01 ^A^
PF	1.93 ± 0.2 ^B^	25.92 ± 2.9 ^A^	9.40 ± 0.95 ^B^	0.064 ± 0.011 ^A^

Results presented as mean ± SD. (*n* = 3). Means in the same column followed by different capital letters (A or B) are significantly different by *t*-test (*p* = 0.03). WVP: Water vapor permeability; Ps: Puncture strength; Eb: Elongation at break; FMA: Mucoadhesive Force.

## Data Availability

Not applicable.
